# A review of health promotion funding for older adults in Europe: a cross-country comparison

**DOI:** 10.1186/s12913-016-1515-2

**Published:** 2016-09-05

**Authors:** Jelena Arsenijevic, Wim Groot, Marzena Tambor, Stanislawa Golinowska, Christoph Sowada, Milena Pavlova

**Affiliations:** 1 Department of Health Services Research; CAPHRI, Maastricht University Medical Center, Maastricht, The Netherlands; 2 Faculty of Health, Medicine and Life Sciences, Maastricht University, Maastricht, The Netherlands; 3 Top Institute Evidence-Based Education Research (TIER), Maastricht University, Maastricht, The Netherlands; 4 Faculty of Health Sciences, Department of Health Economics and Social Security, Institute of Public Health, Jagiellonian University Collegium Medicum, Krakow, Poland

**Keywords:** Health promotion, Older adults, Funding, Europe

## Abstract

**Background:**

Health promotion interventions for older adults are important as they can decrease the onset and evolution of diseases and thus can reduce the medical costs related to those diseases. However, there is no comparative evidence on how those interventions are funded in European countries. The aim of this study is to explore the funding of health promotion interventions in general and health promotion interventions for older adults in particular in European countries.

**Method:**

We use desk research to identify relevant sources of information such as official national documents, international databases and scientific articles. Fora descriptive overview on how health promotion is funded, we focus on three dimensions: who is funding health promotion, what are the contribution mechanisms and who are the collecting agents. In addition to general information on funding of health promotion, we explore how programs on health promotion for older population groups are funded.

**Results:**

There is a great diversity in funding of health promotion in European countries. Although public sources (tax and social health insurance revenues) are still most often used, other mechanisms of funding such as private donations or European funds are also common. Furthermore, there is no clear pattern in the funding of health promotion for different population groups. This is of particular importance for health promotion for older adults where information is limited across European countries.

**Conclusions:**

This study provides an overview of funding of health promotion interventions in European countries. The main obstacles for funding health promotion interventions are lack of information and the fragmentation in the funding of health promotion interventions for older adults.

## Background

Health promotion interventions are seen by some as a tool to improve health and to decrease medical costs [1]. In an aging population, health promotion may not only prevent the onset of diseases and reduce the medical costs related to these diseases but it may also positively affect the evolution of (chronic) diseases and increase active participation of older adults in society [1, 2]. In this way, health promotion may save costs for society in general [3]. For example, some health promotion interventions, such as physical activity programs provided by employers during or outside work hours, promote labor force participation among older adults [4]. Such interventions enable older adults to participate in society and may reduce the burden on the social benefits system [5].

Although health promotion for older population groups may be a valuable investment, there is no clear evidence about how it is funded [6]. In general, health promotion is considered a public good and it is usually funded by revenues from general taxation (including regional and local taxes) [1]. However, recent studies show that resources available from general taxation are not always successfully invested in general health promotion interventions [7]. Specifically, resources that governments aim to spend on health promotion can be re-allocated to other issue-based public health activities [7]. Also, recent studies show that differences in funding of general health promotion are observed between countries, including differences in the mechanisms of resource collection and resource allocation [8]. In some countries, like Austria and France, where the funding of the health care system is based on social insurance contributions, there are attempts to include all health promotion in the insurance packages but those attempts have not been completely successful [9, 10]. In some other countries the lack of resources prevents the inclusion of general health promotion in the insurance package, so health promotion interventions are funded by donations and private sources [8]. Furthermore, health promotion includes a broad scope of activities, some of which are often not considered as a part of the health care system but are rather seen as multi sector activities [7]. Some of those general health promotion interventions are community based or related to the education system [11]. Although they do address public health problems it is considered that they should be funded by the Ministry of Education or by private funding (out-of-pocket payments) [7]. This is also a reason why initiatives to include all health promotion interventions in health insurance packages have been generally unsuccessful [7].

Similar findings are also observed for health promotion interventions for the elderly. The evidence shows that health promotion interventions for older people are frequently multi-sector activities that are funded through general taxation but also through health insurance contributions (resources provided by social or private/voluntary insurance premiums), by resources obtained from NGOs, EU projects and users’ private payments (co-payments additional to insurance premiums or full market-price payments) [8, 10]. As populations are aging, the number of health promotion programs targeting older adults is growing [8]. They are mostly focused on a healthy life-style, mental health or injury prevention among older adults [8]. Frequently within one program it is possible to combine two or more interventions, for example mental health promotion with promotion of labor participation among elderly. Those programs are not only multi-sector activities but they are often multi-country activities [8]. This means that the same program can be conducted in different countries at the same time. The multi-sector and multi-country characteristics imply a great cross-country diversity in funding the health promotion programs for older adults.

Furthermore, the resources allocated to all health promotion interventions are relatively small [12]. For example, OECD countries report that they spend on average 3.1 % of their public health expenditure on health promotion in general [13]. Only a small share of the general health promotion resources are used to fund health promotion for older population groups [7, 8, 12]. Even with an ageing population, priority is frequently given to health promotion for the young. This is motivated by observing that the returns of the investment manifest themselves after a longer period of time and health promotion is therefore more effective when the investment is made at a younger age [1]. This diminishes the resources allocated to the funding of health promotion interventions for older population groups.

Aging populations and scarcity of resources are the main challenges in the funding health promotion interventions for older population groups [2, 12]. Although the challenges are identified, there is no overview of how health promotion interventions for the older adults are actually funded in European countries and how existing methods of funding can contribute to sustainable health promotion interventions for the older adults. Previous reports on funding of health promotion in Europe have not included all countries but only provide general and limited information about funding [8, 14]. A comprehensive overview is necessary to identify good practices and help policy makers to improve the funding of health promotion in their countries by learning from the experience of others [8]. An overview of health promotion funding can also help health professionals to better use the existing models of funding for health promotion interventions [15]. Specifically, health professionals can learn how to better use the existing resources. Furthermore, there are a growing number of health promotion programs for older adults. Although evidence about the effectiveness of those programs is limited, some sources emphasize the importance of those programs for the health of older adults [8]. Furthermore, those programs show how health promotion interventions are funded in practice in different countries. Based on the overview of the funding we will discuss whether it is possible to identify successful examples.

The aim of this study is to explore the funding of health promotion interventions in general and health promotion interventions for older adults in particular in European countries. We also provide information on how selected health promotion programs for older adults are funded in Europe. For the purpose of this study we use desk research to identify relevant information based on official national reports, international databases and scientific articles related to funding of health promotion.

## Methods

We focus on health promotion interventions such as the promotion of a healthy life style (smoking prevention, prevention of alcohol consumption, promotion of physical activities and promotion of healthy eating), primary prevention activities related to mental health and general well-being, fall and injury prevention as well as promotion of labor force participation among non-retired older adults. Our focus is on these particular interventions since they are most frequently reported in European countries [8]. We do not include secondary prevention activities related to the detection of diseases such as screening tests, as well as primary prevention activities related to vaccinations. Also, we do not include tertiary prevention activities that target older population groups already diagnosed with certain diseases, for example health promotion interventions for older adults diagnosed with diabetes mellitus type 2.

For a descriptive overview of how general health promotion interventions and health promotion for older adults are funded in European countries, we focus on functions proposed as descriptive tools for analyzing the funding mechanism of health care systems in general [16]. Those functions include the collection of funds, pooling of funds, allocation of resources and purchasing of services. Based on these functions, we focus on the following aspects of funding: *what are the mechanisms of collecting funds* (general taxation, indirect taxes, earmarked taxes, social insurance contributions, private insurance contributions, out-of-pocket patient payments and other funding like funding from NGOs or EU), *who are the collecting agents* (government, local municipalities, independent public bodies (specialized funds) or providers), and *who is funding health promotion,* i.e. *allocating funds and purchasing services* (federal, regional or local government, insurance companies, EU institutions, NGOs or private institutions). We are aware that within each country, different mechanisms of funding and different funding and collecting agents co-exist and can be combined. In some countries collecting, pooling and funding agents can represent the same institution, while in others a distinction is made. Also, multiple mechanisms of funding can be used within the same country. Based on these three dimensions, we present data for 27 European countries. Although the aim of this study is to provide an overview of funding of health promotion in general and specifically for older adults in EU, information for some countries, to the best of our knowledge, was not available or only limited available in English. Those countries include: Latvia, Luxembourg, Malta and Romania.

Furthermore, for clarification we divide the funding sources in three different categories: *public funding* (taxes and social insurance contributions)*, private funding* (private insurance contribution, out-of-pocket payments, employers) and *others funding* (from international organizations, EU funds, NGOs funds or funds from foreign governments). We make a distinction between health promotion funding in general and funding of health promotion interventions for older population groups.

To search for relevant information, we use different sources of information such as scientific papers, reports, policy documents and documents coming from international organizations, and the following key words: *health promotion, funding (but also financing, costs, coverage), older adults (elderly, older population groups), Europe (but also the country names)*. We use different combination of key words in searching for scientific articles in PubMed, Google Scholar and the NHS Economic Evaluation Database. Furthermore, we use the same key words to search through the databases and reports by international institutions (OECD, WHO, EU) as well as the websites of national and international projects. We focus on English language documents, but when possible, we also include documents in national languages. This was done for the following countries Austria, Bulgaria, Croatia, Germany, the Netherlands, Poland, and Switzerland. Based on the relevant documents (16 research papers and 48 policy papers, documents and reports), we provide an overview of how general health promotion interventions and health promotion for older adults are funded in different countries based on the three questions presented above. We also provide information to what extent health promotion interventions are funded through public, private or other sources. The results are presented in a narrative form complemented by descriptive tables.

We have also searched the WHO library, OECD library, PubMed, and different project databases such as the Vintage project database, the Health and Aging Project (HALE) database, the Health Pro Elderly project database, the AGE platform Europe database, the European network for mental health promotion database (the ProMenPol Database), European network for work promotion database, the National Institute for Public Health Netherlands database, the EuroHealthNet database and the EUNAAPA project database, to identify programs that address health promotion interventions for older population groups. As indicated above, we focus on programs that address a healthy life style, primary prevention activities related to mental health and general well-being, fall and injury prevention and promotion of labor participation among non-retired older adults. We include programs that provide information about funding (who is funding and how) and who is the main program provider. Again, the results are presented in a narrative form complemented by descriptive tables.

## Results

In Table 1, we present our findings on how general health promotion interventions and health promotion for older adults are funded following the three dimensions outlined in the method section. In the majority of the countries the agent that collects resources is also one of the agents that fund the general health promotion programs for example the government in Bulgaria or social insurance in France. While the agents that collect resources include usually one or two governmental bodies, the numbers of agents that fund general health promotion programs are higher and more heterogeneous. Overall, the main agents that collect resources and fund programs are governmental institutions, but funding is also done by private companies, NGOs and EU projects. In countries like Austria, Denmark, Germany, Hungary, Ireland and Switzerland, special funds are created to collect and allocate resources to providers of general health promotion interventions. Resources are usually collected via general taxes and are then allocated to those funds. In Switzerland the resources collected through taxes are combined with private mechanisms of collecting funds, i.e. each person contributes to the insurance general health promotion fund by regular monthly payments.

**Table 1:** Funding of health promotion interventions in European countries

Country	Who is funding health promotion interventions in general?	Who is funding health promotion interventions for older adults?	What are the mechanisms of funding?	Who is the collecting agent?	Sources
Austria	Government Social Insurance fund NGOs^a^ EU funding	Health promotion for older population groups are also funded by Fund for Healthy Austria and health insurance funds. For individuals who use health promotion activities, they are covered by health insurance package(Article 154b, ASVG)	General taxes Insurance contributions	Fund for Healthy Austria 9 regional health insurers 6 professional health insurers	Hofmarcher et al. (2006) [26] Schang LK, et al. (2012) [12].
Belgium	Regional and local entities	Same as general health promotion	General taxes Local taxes Earmarked taxes	Government Local communities	Gerkens S, et al. (2010) [18]
Bulgaria	Government Social insurance fund EU projects	There is a National Plan to Promote Active Aging among Elderly in Bulgaria (2012-2030) adopted through Protocol № 24.2 of the Council of Ministers on 20.06.2012. The objectives of the plan are to promote active aging among the elderly and to develop long-term care and voluntary work directed at the needs of elderly people. The funding of this plan comes from the state budget.	General taxes Private insurance contributions Grants (EU projects)	Ministry of health National health insurance fund	http://journal.frontiersin.org/article/10.3389/fpubh.2015.00175/full http://www.insurancebulgaria.com/health-insurance-package-health-improvement-and-disease-prevention http://www.chrodis.eu/wp-content/uploads/2014/10/JA-CHRODIS_Bulgaria-country-review-in-the-field-of-health-promtion-and-primary-prevention.pdf http://www.hspm.org/countries/bulgaria22042013/livinghit.aspx?Section=3.3%20Overview%20of%20the%20statutory%20financing%20system&Type=Section
Croatia	Government Social insurance fund	Same as general health promotion	General taxes Insurance contributions	Croatian Insurance Fund	Vulic & Healy (1999) [27]
Cyprus	Ministry of Health Different private stakeholders	Same as general health promotion	General taxes Private contributions	Government	http://www.chrodis.eu/wp-content/uploads/2014/10/JA-CHRODIS_Cyprus-country-review-in-the-field-of-health-promtion-and-primary-prevention.pdf
Czech Republic	Ministry of health NGO EU projects	Same as for general health promotion	General taxes Private contributions Grants (EU projects)	Ministry of Health	Bryndová et al (2009) [28]
Denmark	Government Private stakeholders	Same as for general health promotion	General taxes Private insurance contributions Private payments	Government	Christiansen (2002) [29]
Estonia	Estonian Insurance fund European social funding EU projects	Same as for general health promotion	Insurance contributions Grants	Estonian Insurance fund	http://www.chrodis.eu/wp-content/uploads/2014/10/JA-CHRODIS_Estonia-country-review-in-the-field-of-health-promtion-and-primary-prevention.pdf http://programs.jointlearningnetwork.org/content/estonian-health-insurance-fund
Finland	Municipality entities	Financed by municipalities	General taxation Local taxes	Local municipalities	World Health Organization. (2002) [30]
France	Insurance funds	Same as for general health promotion	Insurance contributions Earmarked taxes Taxes on alcohol and tobacco products	Social insurance funds	Fund (2012) [31]
Germany	Statutory health insurance funds Ministry of Health, Labor, Family and Social affairs Federal Association for Prevention and Health Promotion Local communities State Associations for Health Promotion and Prevention; Private insurance funds Financial resources from foundations (e.g. Robert Bosch Foundation, Bertelsmann Foundation)	Same as for general health promotion	General taxes Social insurance fund Private households Workers payments Donations	Social insurance fund	Prävention und Gesundheitsförderung weiterentwickeln. Positionspapier des GKV-Spitzenverbandesbeschlossen vom Verwaltungsrat am 27. Juni 2013 https://www.gkv-spitzenverband.de/media/dokumente/krankenversicherung_1/praevention__selbsthilfe__beratung/praevention/2013-07-11_Positionspapier_Praevention_und_Gesundheit.pdf Brussig (2014) [32] Conflicting Rules and Incentives for Health Promotion and Prevention in the German Statutory Health Insurance (GKV). Health promotion effectiveness: testing the German statutory health insurance agencies evaluation system in health promotion, and preliminary findings from 212 health training courses
Greece	Government EU funding	Same as for general health promotion	General taxation Insurance contributions Grants	Government	http://www.chrodis.eu/wp-content/uploads/2014/10/JA-CHRODIS_Greece-country-review-in-the-field-of-health-promtion-and-primary-prevention.pdf http://www.ep.liu.se/ej/hygiea/v9/i1/a18/hygiea10v9i1a18.pdf http://www.euro.who.int/__data/assets/pdf_file/0004/130729/e94660.pdf
Hungary	Government Health fund for health promotion	Same as for general health promotion	General taxes	There is a special fund for HEALTH PROMOTION financing	Schang LK, et al. (2012) [12]
Ireland	Healthy Ireland Fund Local communities	Same as for general health promotion	General taxes Social insurance contributions Private insurance Pit of pocket patient payments	Healthy Ireland Fund	What works in health promotion for older people? NATIONAL COUNCIL ON AGEING AND OLDER PEOPLE 22 CLANWILLLIAM SQUARE GRAND CANAL QUAY DUBLIN 2, report
Italy	Government	Same as for general health promotion	Tax based	Government	Fund (2012) [31]
Iceland	Government EU projects NGOs	Same as for general health promotion	General taxes Grants	Government	Fund (2012) [31] http://www.chrodis.eu/wp-content/uploads/2015/02/Italy-CHRODIS-final-draft_rivistoBD_DG.pdf
Lithuania	Government Insurance fund	Same as for general health promotion	General taxes Insurance contributions	Insurance fund	http://www.mepactiveageing.ipleiria.pt/files/2012/01/Klaipeda-State-College1.pdf
The Netherlands	Government NGOs	Government	Local taxes Private payments	Government	http://www.nationaalkompas.nl/preventie/gericht-op-doelgroepen/ouderen/ Schippers et al (2009) [33]. http://www.rivm.nl/bibliotheek/rapporten/270102001.pdf http://www.healthproelderly.com/pdf/National_report1_Netherlands.pdf
Norway	Organized and covered by municipalities via general taxes. Some funds are obtained also via Norwegian Health Economics Administration fund	Same as for general health promotion	Local taxes Private payments	Government	Thomson et al (2011) [34]
Poland	Government Regional entities Local communities National insurance fund NGOs	Same as for general health promotion	General taxes Earmarked taxes Social insurance contribution	Governments Territorial self-government National insurance fund	Izabela Nawrolska (2013) [35]
Slovakia	Government	Same as for general health promotion	General tax Social insurance Users payments	Government	Colombo and Tapay (2004) [36]
Slovenia	Insurance funds NGOs EU funding	Same as for general health promotion	Voluntary health care insurance contributions Grants Donations	Insurance fund Specialized fund for health promotion	Jakubowski (Ed.) (2002) [37]
Spain	Government Ministry of Health	Same as for general health promotion	General taxes	Insurance fund	World Health Organization. (2000) [38]
Sweden	Included in universal coverage	Spare evidence of users payments for older population groups	General taxes	Insurance fund	Care of the Elderly in Sweden Today
Switzerland	Insurance funds	Users payments exists among older population groups	Insurance contributions Private payments	Fund for health promotion Gesundheitsförderung Schweiz GFS	Gesundheitsförderung Schweiz, Geschäftbericht e.g. 2013 under: http://geschaeftsberichte.gesundheitsfoerderung.ch/2013/
United Kingdom	NHS	Users payments exists among older population groups	Same as general health promotion	Covered by NHS Financed by government or charity organizations or private payments	Courbage and Coulon (2004) [19]. Ashton (2001) [39]

^a^NGOs in Austria also receive money from general taxation

Our results also show that general taxes are the main mechanisms to collect funds. However, other mechanisms are also observed and very often combined with each other. In countries such as Belgium, France and Iceland, general health promotion interventions and health promotion for older adults are funded by a combination of social insurance premiums, general and earmarked taxes (taxes on alcohol or tobacco products) [17, 18]. However, funding via private insurance in combination with other mechanisms of collecting funds is not common (except in Switzerland and Slovenia). General health promotion interventions and health promotion for older adults are sometimes also funded by international projects and local NGOs. NGO donations and EU funding are most often reported in Croatia, Estonia, Lithuania and Slovakia. In those countries public funding is coming from social insurance premiums or general taxes via the Ministry of Health, while EU funding is mostly related to European Commission projects. In the UK, general health promotion and health promotion for older adults are funded through the National Health Service (NHS), but also through charity organizations and private insurance funds [14, 19].

In the Netherlands, general health promotion interventions and health promotion for older adults are funded by local and general taxation and the government is the main funding agent, in particular the Ministry of Health. The main funding agents allocate resources to different institutions such as local communities, the TRIMBOS institute or RIVM. Also, in the Netherlands there is a public-private mix of health promotion funding. An example is the GALM (Groningen Active Living Model) program where 50 % of the funding is received from the government, while additional resources are provided by private stakeholders and patient co-payments [8]. Another example is the Nationaal Programma Ouderenzorg (National Program Elderly Care, NPO) that includes a large number of health promotion projects for older adults organized through eight regional organizations that cover the whole country that are funded through general taxation, private organizations and private user’s payment [20]. In this case, different funding agents and different mechanisms of collecting funds are used within the same country.

Another interesting case, where different mechanism of collecting funds and different funding agents are used within same country is Germany. The dominant source of general health promotion funding is the statutory social health insurance (Gesetzliche Krankenversicherung). It provided 51 % of all funds available for health promotion in general in 2013. The second most important sources are private household resources and funds from NGOs. It is estimated that 19 % of the total amount available for health promotion is coming from those sources. The third group is resources from employers who provide 15 % of the total amount related to general health promotion and the fourth group comprises resources form government budgets with a contribution of 13.4 %. In this way Germany combines public, NGOs and private methods of funding general health promotion interventions.

If we combine the main funding agent with the most often used mechanisms of funding, we see that in the majority of countries, the main funding agents are government institutions and insurance funds while the main mechanism of collecting funds is general taxation. This includes countries like Bulgaria, Greece, Finland, Iceland, Italy, Norway, Poland, Portugal, Spain and Sweden.

If we combine the main mechanism of collecting funds (via general taxation and different funding) and collecting agents, we observe diversity among European countries. For example, in Norway and Finland general health promotion interventions and health promotion for older adults are funded by local communities that collect resources via general taxes, while in Sweden, resources collected by general taxes are allocated through the universal health insurance agency. In this way, general health promotion interventions in Sweden are part of the universal health care coverage. In Poland resources are collected by general taxes but can be allocated by local and regional authorities. However, evidence shows that in most countries where the government is the main agent of funding and where mechanisms of collecting resources is dominated by general taxation, there are also private and external funding agents, mostly NGOs and private companies via donations.

Only few European countries such as Germany, Finland, Iceland, the Netherlands, Norway and Sweden have specific budget line in their national budget for funding general health promotion.

In Table 2, we show to which extent public, private and others funding (those coming from NGOs and EU projects) are combined in different countries. Although general health promotion interventions are funded mostly by public internal funding, there is a significant number of countries where public funding is combined with external sources (7/27). Public funding is also combined with private sources and this is the case in seven countries (Denmark, Germany, the Netherlands, Norway, Slovenia, Switzerland and the UK).

**Table 2:** Funding of health promotion activities based on type of sources

Country	Type of sources for funding health promotion
Austria	Public and others sources
Belgium	Public sources
Bulgaria	Public and other sources
Croatia	Public and others sources
Cyprus	Public sources
Czech Republic	Public sources
Denmark	Public and others sources
Estonia	Public and others sources
Finland	Public sources
France	Public sources
Germany	Public private and others sources
Greece	Public sources
Hungary	Public sources
Ireland	Public and private sources
Italy	Public sources
Iceland	Public sources
Lithuania	Public and others sources
The Netherlands	Public, others and private sources
Norway	Public, others and private sources
Poland	Public and others sources
Portugal	Public
Slovakia	Public and others sources
Slovenia	Public, others and private sources
Spain	Public sources
Sweden	Public sources
Switzerland	Public and private sources
United Kingdom	Public and private sources

In Table 3, we present selected programs on health promotion for older population groups and their funding. We identified 98 different programs. The majority of the programs for older adults are funded by public sources. In some countries (Finland, Denmark), the government is directly involved in funding. In other countries, the Ministry of Health is the main agent of funding (21.6 % of all programs in our sample are funded directly by the Ministry of Health). Programs funded by the EU fall within the framework of cooperation between countries, while two programs are jointly funded by governments of two neighboring countries, i.e. a program for social networking among older population groups in Poland funded by the German and Polish government and a program for mental health prevention funded by the government of Slovenia and Hungary.

**Table 3:** Funding of programs related to health promotion interventions for older population groups

Country	Name of the program	Type of activity	Target group	Who is provider	Funding
Austria	Kleeblatt	Diet, exercise, motivation, social life	General	Public non-profit organization	Fonds Gesundes Österreich Fonds Gesundes Vorarlberg
Austria	“Happy together” – Fitness and nutrition courses for migrants from Turkey	Fitness, nutrition	Educationally disadvantaged older people Older people from minority ethnic groups Older women Socio-economically disadvantaged older people	Public non-profit organization	Fonds Gesundes Österreich Fonds Soziales Wien Wiener Krankenanstaltenverbund
Austria, Germany, Italy, Lithuania, UK	SenEmpower	Self-employment	Older adults	EU funds	Life Long learning programs EU
Austria	Aktiver Lebensabend	Active-retirement	Older adults	Public non-profit organization	City of Graz
Austria	Moving stories	Story and theater in nursing homes	Older adults	Public non profit	Health fund austria
Austria	Health of the elderly generation	-	Older adults	Public non-profit	Bundesministerium für Gesundheit
Austria	Plan60 – Health promotion for older people in urban areas	Social inclusion, Better quality of life	Older than 60	Public non-profit	Fund for healthy Austria
Austria	Changing Track at Third Age	Social inclusion	Older women	Public non-profit	European Commission Austrian statutory cooperation Own funding
Austria	Active Ageing! Investment in the health of older people	Social inclusion Health education	Minorities	Public non-profit	World Health Organization (WHO) Fonds Soziales Wien
Austria	The spider and the net	Social inclusion	Older women-caregivers	Public non-profit	City of Graz (finished)
Austria	Staying mobile for life	Physical and mental fitness	Older adults	Public non-profit	The Federal State of Vorarlberg Material support by the cities and other sponsor(ongoing)
Austria	Ripe Apples	Healthy life style	Older adults	Public non-profit	Federal Ministry for Education, Science and Culture, Fund for a Healthy Austria, City of Graz (finished)
Austria	Promoting Healthy Ageing in Rural and Semi-Urban Communities in Austria	Social networking	Older adults	Public non-profit	Fund for a Healthy Austria finished
Austria	Productive Ageing in the GiroCredit Bank	Age friendly working environment	Older adults	Private profit	Bank-ongoing
Austria	Women’s Autumn	Healthy aging	Older women	Public non-profit	Fund for healthy Austria
Austria	Counselling at the Street Corner	Information about healthy aging	Older migrants	Public non-profit	Federal State of Vienna-ongoing
Austria	LENA - Learning in post-professional phase	Learning in older age	Older adults	Public non-profit	EU Commission
Austria	LIMA – Life Quality in old age	Mental trainings	Older adults	Public non-profit	Fund for healthy Austria
Austria, Germany, Italy, Portugal, UK	LISA – Learning in Senior Age	Education for elderly	Older adults	Public non-profit	EU Commission-government co-funding
Austria	Schmid Skrew Factory	Factory for elderly	Old workers	Private profit	The project is part of the LIFE-Programme of the voestalpine company-ongoing
Austria	SMZ Liebenau – Seniors platform	Social networks	Older adults	Public non-profit	Sozialmedizinisches Zentrum (SMZ) Liebenau
Netherlands	Pink buddies	Loneliness, depression	Older -homosexual	NGOs	The Schorer Foundation receives financial support for their projects from private funds, local authorities and sponsors.
Netherlands	GALM/Groningen Active Living Model	Physical activities	Older adults	NGOs, public	The government contributes 50 % to a local project on the basis of the so-called ‘Breedte Sport Impuls’, a financial regulation encouraging sports activities. Participants contribute about € 2.50-€ 3.00 per person.
Netherlands	Activating home visits for and by elderly immigrants	Social-emotional support	Older adults	Public non profit	ZonMw
Netherlands	Friendship enrichment programme for older women	Social inclusion	Older women	Public non profit	ZonMw
Netherlands	GRIP on life: a Bibliotherapy in Self-Management Ability (SMA)	Self-Management Ability (SMA)	Older adults	Public non profit	ZonMw
Netherlands	The course ‘Looking for meaning in life’	Decrease depression	Older adults	Public non profit	Trimbos Institute (Dutch Institute for Mental Health and Addiction) and ZONMW.
Netherlands	Falling-clinics	Preventing falling	Older adults	Public non profit	General health clinics, medical centres or hospitals
Netherlands	‘Be down and brighten up 55+’	Mental prevention	Old migrants	Public non profit	GGZ-instellingen
9 EU countries	Future Elderly Living Conditions in Europe		Older adults	Public non profit	-
Denmark	Healthy Throughout Life	improving quality of life and reducing social inequality in health.	Older adults	Public non-profit	government
Finland	Quality recommendations for guided health-enhancing physical activity for older people	Physical activity	Older adults	Public non-profit	Government, local municipalities
France	The Elderly	Healthy life style	Older adults	Public non-profit	Government
Hungary	Improving the health of the elderly	Healthy life style	Older adults	Public non-profit	-
Austria, Czech Republic, Germany, Italy, Lithuania, UK	From Isolation to Inclusion	Social inclusion, poverty	Older vulnerable groups	Public non-profit	Second Trans-national Exchange Programme of the European Commission, 2005 – 2007
Sweden, Finland, Poland and UK	Ageless at work	Labor participation	Oder adults	Public non profit	EU Commission; funding instrument ESF
Austria, Bulgaria, Germany, Greece, Hungary, the Netherlands, Slovenia, Switzerland	MATURE@eu	Improve conditions for older workers	Older adults		EU-funding instrument: Leonardo da Vinci
Czech Republic	Older women and mental health promotion	Quality of life of older women, depression, stres	Older women	Private non profit	National Programme on Health - Health Promotion Projects, Ministry of Health of the CR,
Czech Republic	Improvement in the nutrition of older people as a supporting factor of their general health status	Quality of life	Older adults	Public non profit	National Programme on Health - Health Promotion Projects, Ministry of Health of the Czech Republic
Czech Republic	Healthy Aging	Prevention of fall	Older adults in nursing homes	Private non profit	National Programme on Health- Health Promotion Projects, Ministry of Health of the Czech Republic (Národní program zdraví - Projekty podpory zdraví)
Czech Republic	Effect of reminiscence therapy on the health status and quality of life of residents of care homes	loneliness	Older adults in nursing homes	Public non profit	Internal Grant Agency of the Czech Ministry of Health
Czech Republic	No fear from healthy ageing	Physical activity	Older adults (finished)	Public non profit	National Programme for Health- Health Promotion Projects, Ministry of Health of the Czech Republic (Národní program zdraví - Projekty podpory zdraví, Ministerstvo zdravotnictví CR)
Czech Republic	Cognitive training and physical fitness programmes for older people	Prevention of mental health Physical activity	Older adults	Public non profit	National Programme on Health - Health Promotion Projects (Národní program zdraví- Projekty podpory zdraví) - Ministry of Health of the CR Municipal Authority of the City Sokolov
Germany	Fit for 100	Physical activity	Older than 80	Public non profit	Ministry of Labour, Health and Social Affairs North Rhine-Westphalia (MAGS)
Germany	conversation Cafe for Older Citizens of Görlitz	Mental health prevention	Older adults	Public non profit	Insurance companies Private companies
Germany	Aging and Health - Patient Education for Women	Quality of life	Older women migrants	Public non profit	AOK
Austria, Bulgaria, Germany, Greece, Hungary, the Netherlands, Slovenia, Switzerland	MATURE@eu	Improve conditions for older workers	Older adults	Public non profit	EU-funding instrument: Leonardo da Vinci
Italy	Immigration as a social resource, rather than a source of fear	Social inclusion	Older adults	Public	Social Solidarity Ministry
12 EU countries	WeDO2 - For the wellbeing and dignity of older people	Social networks, social inclusion	Older adults	Public	European Commission Lifelong Learning Programme
Sweden, Netherland, Norway, Hungary, Italy, Germany, Ireland	IROHLA - Intervention research on health literacy among the ageing population	Health literacy	Older adults	Public	EU
France, Poland and Ireland	EMIN works on adequacy of minimum old age income schemes	Social inclusion	Older adults	Public	Polish Committee for the Scientific Research
Poland	Older Man, Older Woman	Abuse prevention	Older adults	Public	Funds from local authorities
Poland	Encouraging mutual support amongst older people in Antoniuk in Bialystok	Social support	Older adults	Public	Foundation for Polish-German Cooperation Committee for Scientific Research
Slovakia	I am 65+ and happy to live the healthy life	Quality of life	Older adults	Public	government
Slovakia	Memory training for older people	Mental health	Older adults	Public	Local hospitals
Slovakia	Programmes for active ageing	Social networks	Older adults	Public	Members fees and donations
Slovakia	Seniors, join in	Intergenerational solidarity	Older adults	public	Ministry of Transportation, Post-Office and Telecommunications of the SR
Slovakia	Successful ageing	Mental health prevention	Older adults	Public	government
Slovenia	Career plan for 50+	Labor activity	Older adults	Private	Center for lifelong learning; center for new knowledge
Slovenia	Dancing in old age	Physical activity; social interaction	Older adults	Public profit	City of maribor
Slovenia	Better quality of life for older people	Quality of life	Older adults	public	share CBC, Joint Small Project Fund Slovenia/Hungary 2002.
Slovenia	Foreign languages - University for the third life period	Mental health	Older adults	Public profit	Local communities
Slovenia	Community Nursing Care	Mental health	Older adults	Public	Ministry of Health, National Health Insurance System and local communities, Institute for Health Protection
Slovenia	Intergenerational camps	Solidarity	Older adults	Public	Ministry of Labour, Family and Social Affairs.
Slovenia	Mobility for health	Physical activity	Older adults	Public	Univerza za tretje zivljensko obdobje Bela Krajina
Slovenia	Self-help groups for older people	Social inclusion, mental health	Older adults	Public	Ministry of Labour, Family and Social Affairs; local communities
Spain	Expert Patients	Mental health	Older adults	Private	Consejería de Sanidad de la Región de Murcia
Spain	+ plus life	Cognitive skills	Older adults	Private	FATEC-older people association in catalania
Spain	Community project for falls prevention	Fall prevention	Older adults	Public	ABS Salt-local communities
Spain	Supportive Halls	Solidarity neighbors	Older adults	Private	Obra Social Cajamadrid
Italy	The solidarity project	Social support	Older adults	Public	Advisory to the social politics and health promotion of Rome Municipality donations by TIM society donations by Gemm Spa
Italy	Clowns in health care homes (R.S.A): jocularity therapy	Mental health	Older adults in nursing homes	Private	CADIAI Social Cooperation
Italy	Improving the quality of life in the third age through new technology	Mental health	Older adults	Public	Region of Liguria (regional funds, national, communitary)
Italy	Immigration as a social resource, rather than a source of fear	Social inclusion	Older adults	Private	Social Solidarity Ministry
Spain	Let’s go	Physical activity	Older adults	Private	Spanish red cross
Spain	Ageing School	Emotional support, physical activities	Older adults	Public	Local communities
Spain	Active Company	Walking activities	Older adults	Public	Red cross
UK	Providing health promotion to older people - Suffolk Social Care	Social support	Older adults	Public	West Suffolk Primary Care Trust
UK	A specialist health and social care team for the promotion of health and independence in ‘at risk’ older adults	Social security	Older adults	Public	Camden and Islington Primary Care Groups
UK	Positive Action on Falls: A Peer Education Approach	Fall prevention	Older adults	Public	Department of Trade and Industry (DTI)
UK	Chair Based Exercise Project	Physical activity	Older people	Public	North Yorkshire and York Primary Care Trust
UK	RISE	Social inclusion	Older adults	Private	Regenerate-RISE, Charitable organization
UK	Health promotion through sports and recreational activities	Physical activities	General population/older adults	Private	Local health authority in the North East of England
UK	Bromley-by-Bow Centre	Emotional support	Older adults	Private	Charitable donations
UK	The Forth view Drama Project	Mental health prevention	Residential home	Public	Fife Council
UK	Sharing and Caring	Mental health	Older adults	Private	Age concern
UK	Older Adults Support Service in Southwark (London, UK)	Alcohol prevention	Older adults	Public	government
Italy	Data club project	Alcohol prevention	Older adults	Public	Research body
UK	Alcohol and older people	Alcohol prevention	Older adults	Public	Funded by other sources: ICGP and National Council for ageing and Older People
Germany	Independent in seniority – addiction issues can be solved	Alcohol prevention	Older adults	Public	government
Germany	Health Promotion for Older Migrants - The Göppingen Project	Healthy life style	Older migrants	Public non profit	Ministry of Health and Social Security Citizens Foundation of the City of Göppingen Neue Württembergische Zeitung and Kreissparkasse Göppingen
Germany	Prevention of Falls in Nursing Homes	Fall prevention	Older adults in nursing homes	Public	Federal Association of BKK Working group Ulm Working group Hamburg (finished)
Germany	Campaign Addiction Prophylaxis. Work group older people	Alcohol prevention	Older adults	Private	AOK
Germany	Active Health Promotion in Old Age	Mental health prevention	Older adults	Public	Federal Ministry of Family, Seniors, Women and Youth (BMFSFJ). Foundation Max und Ingeborg Herz.
Germany	Senior Networks of Cologne	Social networks	Older adults	Public	City of Cologne
Germany	Federal Government’s Pilot Project Really fit from 50 onward	Physical activity	Older adults	Public	Ministry of Family Affairs, Senior Citizens, Women and Youth Kneipp Factories
Greece	Implementation of a physical exercise program for third age people in the municipality of Thessaloniki. Four years on: progress, comments, conclusions.	Physical activity	Older adults	Public	Municipality of Thesaloniki
Greece	Action programs for older people	Physical activity	Older adults	Public	Municipality of Agios Dimitrios
Italy	Evaluation of neighborhood assistance for frail older people	Social support	Older adults	Public	Municipality of Brescia City

Nearly one in six (15.5 %) of all programs are funded through specialized funds for health promotion activities. However, in those countries, other agents of funding are also involved, for example local municipalities in Austria and Germany. Programs with private funding (participants and/or private companies) are less often identified (10.4 %). Programs that are funded through a public-private mix represent 10.3 % of the programs in Table 3. Private agents of funding include private companies or participants. For several programs in Germany, the Netherlands and Switzerland participants pay a fee. This is for programs that are partially funded from public sources (public-private mix).

## Discussion and conclusion

Our results illustrate the great diversity in funding of general health promotion and health promotion for older adults across Europe (Table 1). Diversities are observed in the mechanism of collecting funds and the collecting and funding agents. This diversity is not only related to the fact the general health promotion interventions as well as health promotion for older adults are multi-sector activities, but also to the fact that their funding is related to country-specific characteristics such as health care system funding and government organization. For example, general taxation is the most often used mechanism of collecting funds and the government is most often the main agent of funding, but diversities are also observed in this case. In order to secure the funding for multi-sector activities, some governments (Finland, Sweden) include local municipalities as responsible agents for general health promotion and entitle them to use local and general taxation to fund health promotion. Inclusion of local communities as funding agents enable the funding not only for general health promotion interventions related to health care system but also community based interventions [2]. In some other countries, to secure the funding of multi-sector interventions and also to secure the allocation of resources for general health promotion, governments have created specific institutions responsible for health promotion. An example is the Austrian Health Promotion Foundation (FGOE) that particularly aims to secure the allocation of public sources to evidence-based health promotion interventions [21]. In countries like Belgium, France and Iceland earmarked taxes are used for funding general health promotion as well as health promotion for older adults [18, 22]. In Belgium and France earmarked taxes are combined with social insurance premiums, while in Iceland they are combined with local taxes. Earmarked taxes are seen as a financial incentive with a great potential to raise additional resources for health promotion [23]. Nevertheless, they are still not widely applied in Europe [23].

Diversity in funding is observed not only between countries, but also within countries. This is most visible in countries where local communities or regional cantons are the main source of funding. One example is Belgium, where four different regional governments apply different mechanisms to fund general health promotion [18].

Besides general taxation, social insurance premiums and donations from sources such as NGOs or EU projects also play an important role. External funding such as donations from NGOs or EU funds are quite common in Central and Eastern European countries. One of the reasons for this can be the lack of public resources in those countries. Another reason can be that decision makers in those countries know that external funding is available for health promotion and therefore do not allocate public sources to health promotion. Private sources such as private insurance funds, private companies or users are also important but rare actors in funding general health promotion interventions and health promotion for older adults. The limited evidence shows that users’ payments are mostly used as financial incentives to ensure the financial sustainability of health promotion for older population groups. Sometimes, they are also used as an incentive device for users to continue with their activities.

To describe the funding of general health promotion intervention and health promotion for older adults was more difficult than to assess the funding of some other types of health care services. The reason is the lack of detailed data in the literature sources we identified about the scope of the resources invested in health promotion in different countries. Even in databases of the OECD and WHO, there is no specific information on the percentage of public health expenditure on general health promotion in European countries. In some countries, there are estimated data available from national sources [14]. They usually report a percentage of public health expenditure that is spent on general health promotion and prevention [14]. Data related to resources coming from different types of funding such as private contributions or funding from NGOs and EU projects are even more limited. In order to overcome this lack of information, we have created three groups of countries based on the most frequently used type of funding: public, private or others funding (those coming from NGOs and EU funds) (Table 2). Those groups are descriptive and not exclusive; they are rather an attempt to show to what extent public, private or NGOs and EU projects funding are used in different countries. For example, in countries classified as mostly public funding, there are also health promotion interventions that are funded through external or private funding. Although descriptive, those results show the need for more detailed information such as type of resources used for funding and amounts that are invested in the funding of general health promotion. Providing a budget line in governmental budget for funding the health promotion for each target group, can assure the availability of such information.

In order to illustrate how health promotion for older adults is funded in practice, we have analyzed the funding of health promotion programs. The results show that most often programs are funded by both public and private resources (see Table 3). This is in accordance with the results from the desk research presented in Table 1. However, private funding is more often reported when we use the data from the programs (see Table 3), than in the data from the desk research (see Table 1). The reason for this can be the fact that we used only evidence based programs that are available on web-platforms in English. This may exclude national publicly funded programs from our search. The real extent of the programs that address health promotion for older population groups may be broader than this. Another reason can be the fact that privately funded programs may be overlooked in policy documents that focus on publicly funded interventions. Also our results show that the number of programs funded exclusively through EU funding is growing but their sustainability is questionable. Most of those programs are not sustained after the EU projects are finished [8].

This study shows that health promotion interventions, in general and those focusing on older adults in particular are multi-sector activities that can be funded through different agents and mechanisms of funding. Despite the diversity in funding, public funding is the most often used. In the majority of the countries, both funding from NGOs and EU projects and private funding, are seen as additional tools, but not as the main sources of funding. Although the diversity in funding can be seen as a way to generate more resources for health promotion, it can also impose problems in resource allocation [7]. For example, even if EU resources are available, some countries do not use them but rather rely on internal resources [23].

Overall, the great diversity in the funding of health promotion illustrate that there is no “golden standard” within European countries, but that the model for funding the health promotion reflect country specific characteristics. The existence of a specific fund for health promotion interventions in combination with an evidence-based approach may lead to a more effective use of resources. An example is the Austrian Health Promotion Foundation (FGOE) that allocates resources only to evidence based health promotion interventions*.*

However, the main problem in funding health promotion is related to the lack of information regarding the type of resources (public, private or others) and the amounts that are invested in health promotion. Providing a budget line for funding general health promotion with governmental annual budgets can be used to overcome this situation. Furthermore, it is necessary to provide the information not only for funding the health promotion based on type of intervention (mental health promotion, tobacco cessation), but also based on target groups (older adults, vulnerable groups etc.). Such a strategy can increase the transparency in the use of resources and improve sustainability of health promotion interventions.

Our results are in accordance with recently published reports [8, 14]. However, this study goes one step further as we combine different types of sources (documentations, data bases and web-platforms). We have also included most European countries, while previous reports are based on overviews of only 14 countries. Nevertheless, this study has some limitations as well. The main limitation is that the results are mainly based on documents that report information about health promotion intervention in general. Most of the documents are policy papers, project reports or “grey literature”, while the number of scientific articles that on the funding of health promotion is limited. The inclusion of all types of documents in the analyses can increase the validity of the conclusions. Another limitation is that the search strategy for some countries relied on English language documents only. This can also influence the extent to which information is detailed. For some countries, where we were able to rely on national language literature, the number of sources and quality of information were higher. On the other hand, in some other countries using the national language documents did not increase the quality of information*.*

Another obstacle is a lack of information about funding of health promotion interventions for older population groups. The main reason for this is that data regarding the funding of general health promotion are usually reported by the type of activities and not by the target population group. The only exception is younger adults. The lack of clear information on the funding of health promotion for older population is a topic for attention in the future. Even in countries where special institutions to finance health promotion exist, information about the funding of general health promotion is limited. An ageing population accompanied with scarce resources, increases the need for evidence-based and cost effective health promotion interventions.

Despite the limitations mentioned above, this study provides insight in the funding of health promotion in general and for older adults in particular. Our results show that the funding of health promotion interventions is fragmented and includes different funding strategies. Based on the available information, we cannot say what is the “best” way of funding health promotion. If we had more information on the funding of health promotion interventions, we would be able to explore how different mechanisms of funding affect outcomes and whether they can lead to cost savings. Also, this study focuses only on primary health promotion interventions. Some researchers have argued that successful primary health promotion interventions do not contribute to cost savings [24]. They emphasize that the majority of the costs related to older population groups are related to chronic diseases [25]. There is insufficient empirical evidence to support these claims and it is up to future research to examine the relation between the mechanisms of health promotion funding and costs saving for secondary and tertiary health promotion interventions.

This research also gives a broad overview of the extent to which different sources of funding are present in different countries. In some countries general health promotion interventions are dominantly funded by public sources, while in other countries private sources of funding are also used. Whether public sources are spent more effectively than private sources is an issue for future study.
